# Development and validation of a nomogram for early prediction of post-stroke shoulder–hand syndrome: a retrospective cohort study

**DOI:** 10.3389/fnins.2026.1681392

**Published:** 2026-02-09

**Authors:** Xuezheng Li, Yu Min, Lulu Cheng, Yunyun Tao, Meifeng Zheng, Hua Guo, Xuefeng Fu, Lijun Lu, Wen Yang, Hao Li, Zhen Huang, Kaifeng Guo

**Affiliations:** 1 Postgraduate Cultivation Base of Guangzhou University of Chinese Medicine, Panyu Central Hospital, Guangzhou, Guangdong, China; 2 Department of Acupuncture and Moxibustion, Qingdao Central Hospital, University of Health and Rehabilitation Sciences (Qingdao Central Hospital), Qingdao, Shandong, China; 3 Department of Ultrasonography, Panyu Central Hospital, Guangzhou, Guangdong, China; 4 Department of Rehabilitation Medicine, Panyu Central Hospital, Guangzhou, Guangdong, China

**Keywords:** ischemic stroke, model prediction, nomogram, retrospective cohort study, shoulder-hand syndrome

## Abstract

**Background:**

Shoulder-hand syndrome (SHS) is a prevalent complication following strokes. At present, there is no established and dependable method for early prediction of SHS risk. Thus, we undertook this study to create and validate a nomogram for early prediction of SHS following ischemic stroke (IS), with the aim of informing the development of SHS-specific follow-up protocols in clinical practice.

**Methods:**

We retrospectively collected data on IS patients admitted to the Affiliated Panyu Central Hospital of Guangzhou Medical University from October 1, 2019 to March 31, 2024. The data was randomly split into a training set and a validation set in a 7:3 ratio, and LASSO regression was used to filter the modeling variables. In addition, the consistency index, area under the receiver operating characteristic curve (AUC), and calibration curve were used to verify the accuracy and discriminant power of the nomogram.

**Results:**

A total of 514 patients were enrolled in our study. Significant predictors contained sex, occupation, residence, osteoarthritis, gouty arthritis, myodynamia, heart rate, neutrophils, blood glucose, aspartate aminotransferase, and activated partial thromboplastin time. The AUC for the model constructed on the basis of these predictors was 0.777 (95% CI: 0.727–0.826) in the training set and 0.698 (95% CI: 0.615–0.781) in the validation set.

**Conclusion:**

The nomogram constructed on the basis of common clinical features has a high performance in predicting the occurrence of SHS within 6 months after stroke. It can provide a reference for the development of specific prevention programs during clinical practice.

## 1 Background

Stroke is a serious cerebrovascular disease with high morbidity, disability and mortality (GBD 2016 Stroke Collaborators, 2019). Shoulder-hand syndrome (SHS), a common post-stroke complication (Taylor et al., 2021), is characterized by pain, swelling, and limited mobility of the affected shoulder and hand (Zhang et al., 2021). SHS is highly prevalent within 1–3 months after stroke (Dromerick et al., 2008), with a prevalence of 24–60% (Anwer and Alghadir, 2020). Coincidentally, this is a critical period for functional recovery in stroke patients (Stinear et al., 2020). Therefore, SHS has a significant impact on the daily life and rehabilitation process of patients, often hindering their functional recovery when symptoms are severe.

The pathogenesis of SHS is not fully understood and is generally thought to involve a variety of factors. Brain injury due to stroke causes abnormalities in the nervous system, leading to abnormal perception of pain in the shoulder and hand (Hansson, 2004). Inflammatory response to stroke triggers the release of inflammatory factors, such as IL-6 and TNF-α, which can lead to local tissue inflammation and pain (Heijmans-Antonissen et al., 2006; Huygen et al., 2002). Poor local blood circulation due to stroke causes insufficient nutrients in the tissues, which can result in tissue damage and pain (Iadecola and Anrather, 2011). Loss of motor function after stroke induces stiffness of shoulder and hand joints and muscle atrophy, which causes poor venous and lymphatic return, edema and pain in the upper extremities (Lee et al., 2021). Given the unclear pathogenesis of SHS, it is challenging to develop efficient prevention strategies.

Only one study has specifically constructed a predictive model for SHS, but the number of medical records was very small (Yu et al., 2023). Most studies focus only on risk factors for SHS (Birklein et al., 2018; Ganty and Chawla, 2013). This poses a challenge for the early detection of SHS and the production of specific prevention strategies in clinical practice. Hence, the aim of this study was to develop an accurate and personalized predictive nomogram to help clinicians identify high-risk populations for SHS, implement early intervention, and ultimately improve patient prognosis.

## 2 Methods

### 2.1 Study design and patient selection

This study retrospectively collected data on ischemic stroke (IS) patients admitted to the Rehabilitation Medicine Department of the Affiliated Panyu Central Hospital of Guangzhou Medical University from October 1, 2019 to March 31, 2024. All participants met WHO’s IS diagnostic criteria (Vereshchagin et al., 1989). The diagnostic criteria for SHS were based on the Budapest criteria developed by the International Association for the Study of Pain (IASP) (Harden et al., 2010). Exclusion criteria included (i) diagnosis of transient ischemic attack; (ii) combination of traumatic brain injury or cerebral hemorrhage; (iii) combination of acute myocardial infarction and heart failure (HF); (iv) subjects with severe hepatic or renal insufficiency; (v) suffering from combined immune system disorders, hematological disorders, and neoplasms; (vi) presence of fever, infection of the lungs or other parts of the body; (vii) recent history of major trauma or surgery; (viii) incomplete medical history.

### 2.2 Sample size estimation

We used the events per variable principle (i.e., the number of cases of SHS in the training set is more than 15 times that of the final model variable included) to evaluate the number of cases (Peduzzi et al., 1996). Assuming that 10 variables were ultimately included in the predictive model, a minimum of 150 cases with post-stroke SHS would be required in the training set. According to the preliminary research, the prevalence of post-stroke SHS in our hospital was about 40%, and hence the training set required 150/0.4 = 375 cases. In addition, the validation set of the model needed to be considered, and the validation set was assumed to have 125 cases, with 75% for training and 25% for validation. Ultimately, more than 500 cases were needed.

### 2.3 Data collection

Demographic and clinical information was collected from patients at the time of admission, including case number, name, age, sex, body mass index, insurance, occupation, residence, smoking status, smoking years, drinking status, drinking years, hypertension, diabetes, hyperlipidemia, coronary heart disease, HF, previous acute coronary syndromes, history of previous stroke, osteoporosis, osteoarthritis, gouty arthritis (GA), hyperhomocysteinemia, hyperuricemia, muscle tone, myodynamia, heart rate (HR).

Clinical data and treatment information were collected from the patients at the time of IS onset, including time of onset, middle cerebral artery infarction location, treatment modality of stroke, modified Rankin scale at the time of stroke onset, National Institute of Health Stroke Scale (NIHSS) scores at the time of onset, and NIHSS scores 24 h after receiving treatment.

Laboratory parameters were collected within 24 h of admission, including hemoglobin (g/L), red blood cell (10^12^/L), white blood cell (10^9^/L), neutrophil percentage (NEU) (%), blood platelet count (10^9^/L), K^+^ (mmol/L), Na^+^ (mmol/L), uric acid (UA) (μmol/L), blood urea nitrogen (mmol/L), creatinine (μmol/L), blood glucose (GLU) (mmol/L), alanine aminotransferase (U/L), aspartate aminotransferase (AST) (U/L), plasminogen time (S), activated partial thromboplastin time (APTT) (S), international normalized ratio, fibrinogen (g/L), D-dimer (μg/mL), and homocysteine (μmol/L).

### 2.4 Outcome definition and follow-up

SHS is usually defined as a complex regional pain syndrome type I (CRPS-I) that occurs 1 to 3 months after a stroke (Teasell et al., 2009). Although SHS usually occurs weeks to months after a stroke, one study indicated that it may occur 6 months or even longer afterward (Dijkstra et al., 2003). Therefore, in our study, SHS was defined as complex regional pain in the shoulder and hand occurring within 6 months after stroke. The diagnostic criteria for SHS were based on the Budapest criteria developed by IASP (Harden et al., 2010). Stroke patients were followed up at 3 and 6 months after stroke to record whether they developed SHS. The follow-up assessment was conducted by a researcher who was not involved in the patient’s treatment and remained blinded to the patient’s baseline characteristics and group assignments.

### 2.5 Supplementation of missing values

During the data collection process, a very small number of data points were missing. For these variables, we used multiple imputation to fill in the missing values.

### 2.6 Statistical analysis

Data were statistically analyzed using IBM SPSS 25. Continuous variables with a normal distribution were expressed as mean ± standard deviation, and the comparison between two groups was performed using an independent samples *t*-test. Continuous variables with an abnormal distribution were expressed as median (interquartile range), and the comparison between two groups was performed using a Wilcoxon rank-sum test. Count data were expressed as n (%), and were compared between groups using the chi-square test or Fisher’s exact probability method. *p* < 0.05 indicated that the difference was statistically significant.

The data of this study were divided into the training set and the validation set according to a 7:3 ratio. The training set was used to construct a predictive model. The trained model was then validated in the training and validation sets. Lasso regression was used to screen the variables. We included all 49 variables in the Lasso regression, and the final independent predictors were determined based on the results of five-fold cross-validation. We constructed and validated a diagnostic nomogram of IS patients with concurrent SHS based on these predictors. The area under the receiver operating characteristic (ROC) curve (AUC) was used to assess the discriminative power of the model. Additionally, the optimal Youden index was used to select the optimal cutoff probability. The corresponding sensitivity, specificity, positive predictive value, negative predictive value, positive likelihood ratio, and negative likelihood ratio were calculated. Furthermore, calibration curves were used to assess the calibration accuracy of the model, and decision curves and clinical impact curves were utilized to assess its clinical applicability. Model construction and validation were performed using the glmnet, pROC, mice, readxl, rmda, and rms packages in R4.5.1.

## 3 Results

### 3.1 Basic characteristics of patients

**Figure 1:**
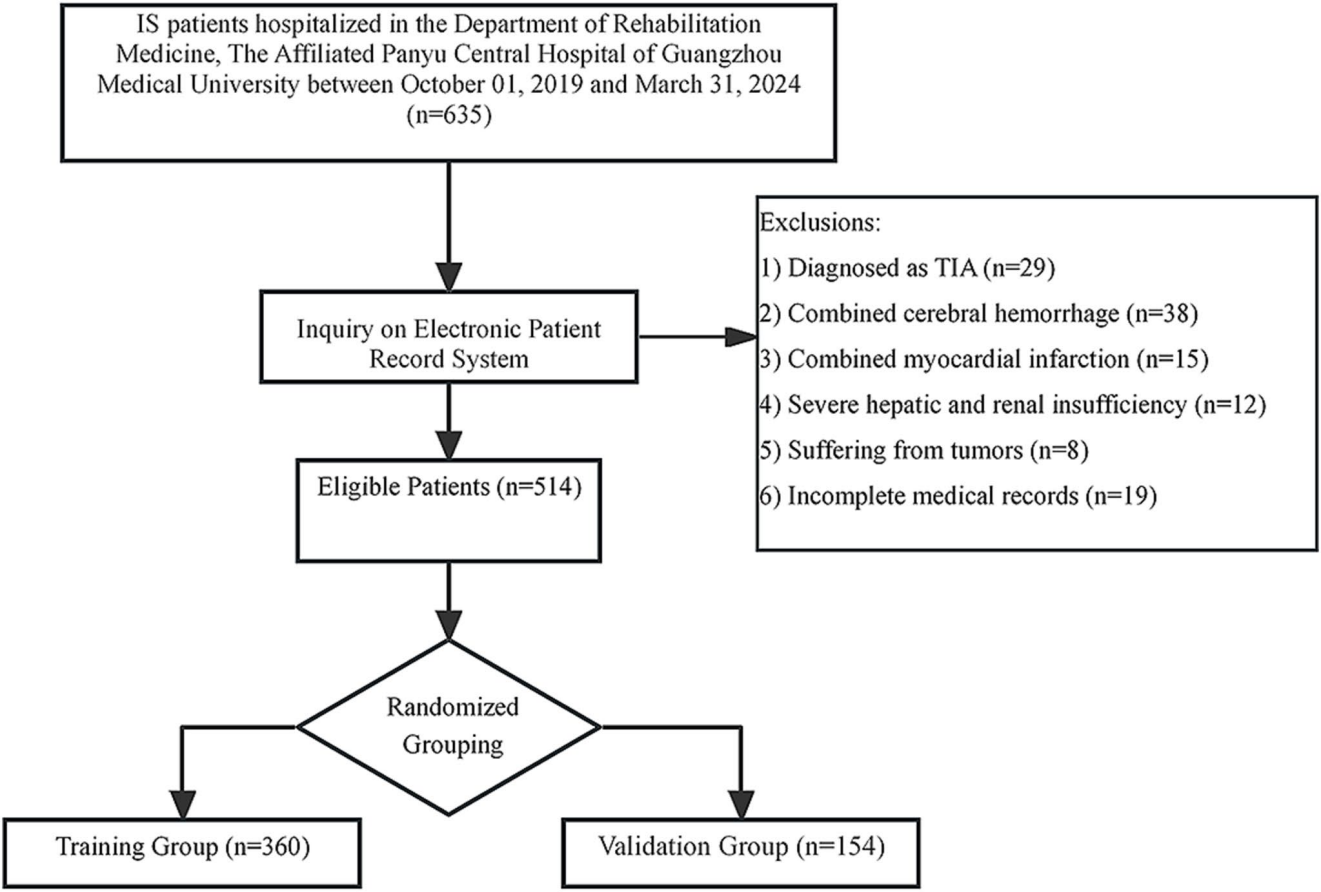
Case screening flowchart. IS, ischemic stroke; TIA, transient ischemic attack.

As shown in the flowchart, between October 2019 and March 2024, 514 patients who met the criteria were enrolled in our study (Figure 1). A small number of variables had missing data, including HF (*n* = 3), years of alcohol consumption (*n* = 5), and history of stroke (*n* = 2). The mean age of the patients was 66.85 ± 11.10 years, with 185 (36%) females and 329 (64%) males. There were 203 (39.5%) patients with SHS. The data was randomly divided into training and validation sets in a 7:3 ratio. Since the dataset was randomly assigned, no formal statistical comparison was performed between the two groups. Detailed basic information is shown in Table 1.

**Table 1:** Basic characteristics of included patients.

Factors	Type	Training (*n* = 360)	Validation (*n* = 154)	Overall (*n* = 514)
Demographics				
Age (years)		67.76 ± 10.99	64.72 ± 11.10	66.85 ± 11.10
BMI (kg/m^2^)		22.73 ± 2.94	22.96 ± 3.25	22.80 ± 3.03
Occupation	Manual worker	121 (33.60)	54 (35.10)	175 (34.00)
	White-collar worker	25 (6.90)	17 (11.00)	42 (8.20)
	Civil servant	19 (5.30)	6 (3.90)	25 (4.90)
	Retired	165 (45.80)	58 (37.70)	223 (43.40)
	Freelance work	30 (8.30)	19 (12.30)	49 (9.50)
Residence	Rural	170 (47.20)	85 (55.20)	255 (49.60)
	Urban	190 (52.80)	69 (44.80)	259 (50.40)
Smoking	Non-smoker	309 (85.80)	136 (88.30)	445 (86.60)
	Smoker	51 (14.20)	18 (11.70)	69 (13.40)
Drinking	Non-drinker	330 (91.70)	143 (92.90)	473 (92.00)
	Drinker	30 (8.30)	11 (7.10)	41 (8.00)
Smoking years		30.00 (20.00)^*^^	28.89 ± 13.67^*^	30.00 (20.00)^*^^
Drinking years		30.00 (30.00)^*^^	27.27 ± 12.72^*^	30.00 (30.00)^*^^
Sex	Female	138 (38.30)	47 (30.50)	185 (36.00)
	Male	222 (61.70)	107 (69.50)	329 (64.00)
Insurance	Rural	149 (41.40)	56 (36.40)	205 (39.90)
	Urban	202 (56.10)	92 (59.70)	294 (57.20)
	Self-paying	9 (2.50)	6 (3.90)	15 (2.90)
Clinical information				
Hypertension	Non-hypertension	52 (14.40)	27 (17.50)	79 (15.40)
	Hypertension	308 (85.60)	127 (82.50)	435 (84.60)
ACS	Non-ACS	339 (94.20)	143 (92.90)	482 (93.80)
	ACS	21 (5.80)	11 (7.10)	32 (6.20)
Diabetes	Non-diabetes	217 (60.30)	91 (59.10)	308 (59.90)
	Diabetes	143 (39.70)	63 (40.90)	206 (40.10)
Hyperlipidemia	Non-hyperlipidemia	256 (71.10)	105 (68.20)	361 (70.20)
	Hyperlipidemia	104 (28.90)	49 (31.80)	153 (29.80)
HR (bpm)		79.90 ± 10.78	80.14 ± 11.24	79.97 ± 10.91
Muscle tone	Normal level	254 (70.60)	116 (75.30)	370 (72.00)
	Reduction	40 (11.10)	11 (7.10)	51 (9.90)
	1–1+	55 (15.30)	22 (14.30)	77 (15.00)
	2–	11 (3.10)	5 (3.20)	16 (3.10)
Myodynamia	0	39 (10.80)	13 (8.40)	52 (10.10)
	<2	66 (18.30)	29 (18.80)	95 (18.50)
	2 or 2 + or 3–	78 (21.70)	32 (20.80)	110 (21.40)
	3 or 3 + or 4–	87 (24.20)	38 (24.70)	125 (24.30)
	≥4	90 (25.00)	42 (27.30)	132 (25.70)
CHD	Non-CHD	329 (91.40)	143 (92.90)	472 (91.80)
	CHD	31 (8.60)	11 (7.10)	42 (8.20)
HF	Non-HF	352 (97.80)	153 (99.40)	505 (98.20)
	HF	8 (2.20)	1 (0.60)	9 (1.80)
StrokeHistory	Absence	296 (82.20)	135 (87.70)	431 (83.90)
	Presence	64 (17.80)	19 (12.30)	83 (16.10)
Osteoporosis	Non-osteoporosis	353 (98.10)	152 (98.70)	505 (98.20)
	Osteoporosis	7 (1.90)	2 (1.30)	9 (1.80)
Osteoarthritis	Non-osteoarthritis	317 (88.10)	138 (89.60)	455 (88.50)
	Osteoarthritis	43 (11.90)	16 (10.40)	59 (11.50)
GA	Non-GA	340 (94.40)	146 (94.80)	486 (94.60)
	GA	20 (5.60)	8 (5.20)	28 (5.40)
Hyperhomocysteinemia	Non-hyperhomocysteinemia	328 (91.10)	141 (91.60)	469 (91.20)
	Hyperhomocysteinemia	32 (8.90)	13 (8.40)	45 (8.80)
Hyperuricemia	Non-hyperuricemia	274 (76.10)	129 (83.80)	403 (78.40)
	Hyperuricemia	86 (23.90)	25 (16.20)	111 (21.60)
Stroke characteristics				
PremRS		3.46 ± 1.04	3.45 ± 1.09	3.46 ± 1.06
PreNIHSS		9.38 ± 6.61	9.39 ± 6.05	9.38 ± 6.44
NIHSS24h		9.10 ± 6.02	8.77 ± 5.37	9.00 ± 5.83
MIL	Cerebral cortex	180 (50.00)	71 (46.10)	251 (48.80)
	Basal ganglia	91 (25.30)	40 (26.00)	131 (25.50)
	Diencephalon	16 (4.40)	6 (3.90)	22 (4.30)
	Brainstem	58 (16.10)	26 (16.90)	84 (16.30)
	Callosum	7 (1.90)	5 (3.20)	12 (2.30)
	Cerebellum	8 (2.20)	6 (3.90)	14 (2.70)
Cure	Conventional medical treatment	266 (73.90)	118 (76.60)	384 (74.70)
	Thrombolysis	53 (14.70)	18 (11.70)	71 (13.80)
	Thrombectomy	41 (11.40)	18 (11.70)	59 (11.50)
Laboratory measures				
HGB (g/L)		125.10 ± 17.65	127.76 ± 19.51	125.90 ± 18.25
RBC (10^12^/L)		4.24 ± 0.59	4.36 ± 0.67	4.27 ± 0.61
WBC (10^9^/L)		6.99 ± 2.09	6.99 ± 1.95	6.99 ± 2.05
NEU (%)		63.14 ± 11.69	61.55 ± 10.59	62.67 ± 11.39
BPC (10^9^/L)		269.06 ± 79.63	267.95 ± 81.16	268.73 ± 80.01
K^+^(mmol/L)		3.85 ± 0.39	3.93 ± 0.35	3.87 ± 0.38
Na^+^(mmol/L)		139.20 ± 2.58	139.10 ± 2.90	139.17 ± 2.68
UA (μmol/L)		346.13 ± 104.48	350.95 ± 97.12	347.57 ± 102.26
BUN (mmol/L)		5.63 ± 2.33	5.56 ± 2.08	5.61 ± 2.26
CRE (μmol/L)		85.31 ± 25.62	84.17 ± 20.08	84.97 ± 24.08
GLU (mmol/L)		5.61 ± 1.48	5.69 ± 1.44	5.64 ± 1.47
ALT (U/L)		25.24 ± 19.90	29.47 ± 26.37	26.51 ± 22.10
AST (U/L)		23.50 ± 10.55	25.62 ± 12.53	24.13 ± 11.21
PT (S)		11.80 ± 3.48	11.57 ± 1.74	11.73 ± 3.07
APTT (S)		27.14 ± 3.18	27.32 ± 2.69	27.19 ± 3.04
INR		1.01 ± 0.17	0.99 ± 0.16	1.00 ± 0.17
FIB (g/L)		3.64 ± 1.32	3.44 ± 0.90	3.58 ± 1.21
Ddimer (μg/mL)		1.01 ± 1.04	0.84 ± 0.75	0.96 ± 0.97
HCY (μmol/L)		11.63 ± 3.95	11.26 ± 3.69	11.52 ± 3.88
Result	Not occurred	226 (62.80)	85 (55.20)	311 (60.50)
	Occurred	134 (37.20)	69 (44.80)	203 (39.50)

BMI, body mass index; mRS, modified Rankin scale; NIHSS, national institute of health stroke scale; HR, heart rate; HGB, hemoglobin; RBC, red blood cell; WBC, white blood cell; NEU, neutrophil percentage; BPC, blood platelet count; UA, uric acid; BUN, blood urea nitrogen; CRE, creatinine; GLU, blood glucose; ALT, alanine aminotransferase; AST, aspartate aminotransferase; PT, plasminogen time; APTT, activated partial thromboplastin time; INR, international normalized ratio; FIB, fibrinogen; HCY, homocysteine; MIL, middle cerebral artery infarction location; ACS, acute coronary syndrome; CHD, coronary heart disease; HF, heart failure; GA, gouty arthritis. *: The years of smoking and drinking are calculated in only smokers and drinkers. ^: Data do not follow a normal distribution; the median (interquartile range) is used.

### 3.2 Differences in various factors in the training set

**Figure 2:**
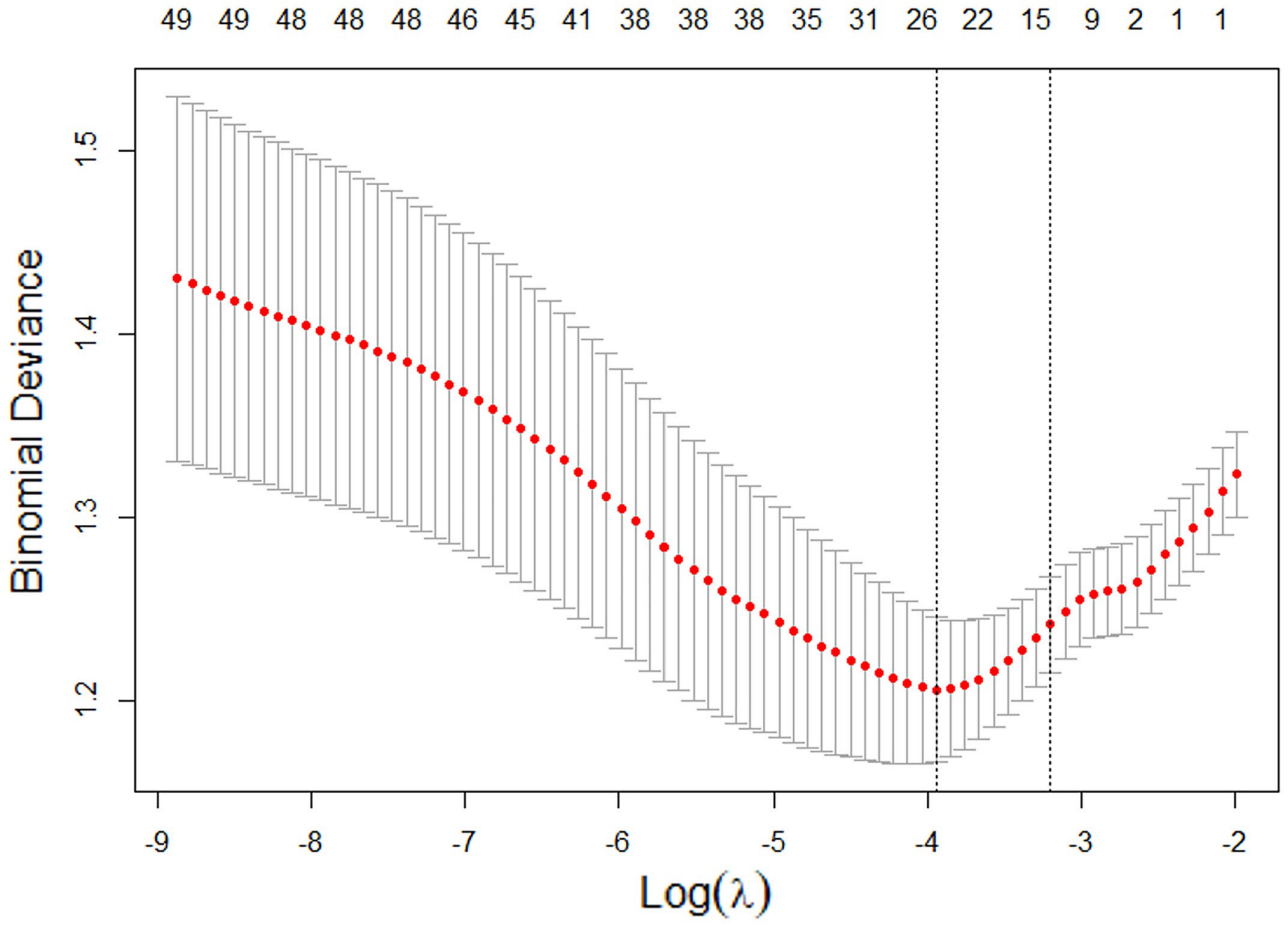
Trends in the number of models with different penalty factors in LASSO regression.

LASSO regression was performed on all modeling variables. The minimum lambda (0.01942) contained 26 variables with non-zero coefficients. However, too many variables could lead to an overly complex model. Therefore, we chose lambda + 1se, which had a value of 0.04087. As shown in Figure 2, 11 factors were shown to be independent predictors of SHS at minimum Log(λ) plus doubled standard error. These 11 factors were sex, occupation, residence, osteoarthritis, GA, myodynamia, HR, NEU, GLU, AST, and APTT. The risk equation is as follows: Logit(P) = 5.793–0.854 × Sex (Male) + 0.581 × Occupation (White-collar) + 0.662 × Occupation (Civil servant) + 0.662 × Occupation(Retired) + 0.662 × Occupation (Freelance) + 0.6321 × Residence (Urban) + 2.1698 × Osteoarthritis (Yes) + 1.7319 × GA (Yes) + 0.0037 × Myodynamia (<2) + 0.0759 × Myodynamia(2 ∼ 3−) − 0.2555 × Myodynamia (3 ∼ 4−) − 0.8468 × Myodynamia (≥4) − 0.0172 × HR − 0.0214 × NEU − 0.2102 × GLU − 0.0325 × AST − 0.0746 × APTT.

### 3.3 Construction of predictive nomogram

**Figure 3:**
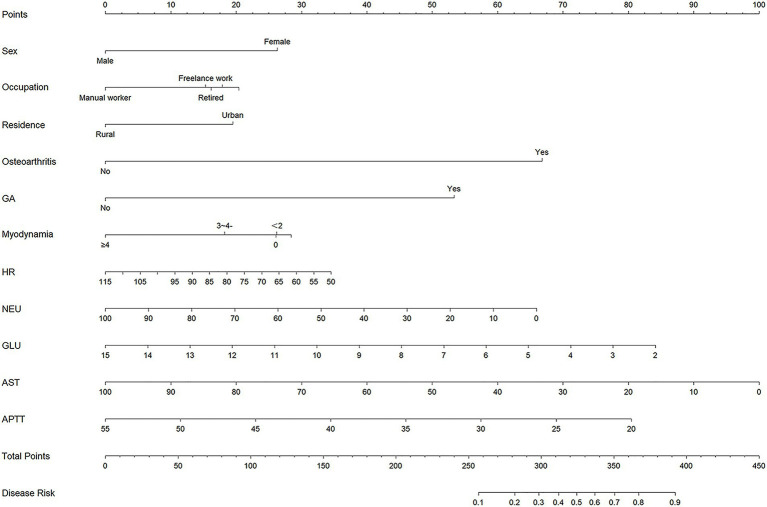
Nomogram for predicting post-stroke shoulder-hand syndrome. GA, gouty arthritis; HR, heart rate; NEU, neutrophil percentage; GLU, blood glucose; AST, aspartate aminotransferase; APTT, activated partial thromboplastin time.

**Figure 4:**
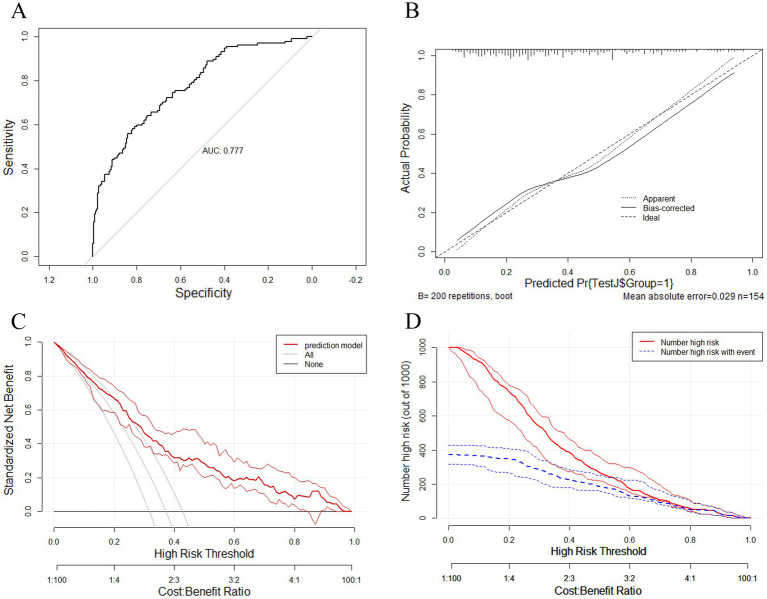
**(A)** Receiver operating characteristic curve of the nomogram for predicting post-stroke shoulder-hand syndrome in the training set. The ROC curve is used to evaluate the model’s ability to distinguish between SHS and non-SHS. A larger area under the curve (AUC) indicates better discrimination. **(B)** Calibration curve of the nomogram for predicting post-stroke shoulder-hand syndrome in the training set. The calibration curve is used to compare the predicted risk with the actual incidence rate. The 45° diagonal line represents perfect calibration. The closer the curve is to the diagonal line, the more consistent the predicted risk is with the actual incidence rate. **(C)** Decision curve of the nomogram for predicting post-stroke shoulder-hand syndrome in the training set. Decision curve analysis (DCA) shows the net benefit under different threshold probabilities. When the model curve is higher than the two reference lines of “treat-all” and “treat-none” within a certain threshold range, it suggests that using the model for decision-making within this range may bring higher net benefits. **(D)** Clinical impact curve of the nomogram for predicting post-stroke shoulder-hand syndrome in the training set. The clinical impact curve shows the number of patients identified as high-risk per 1,000 patients (red line) and the number of patients who actually develop SHS (blue line) at different threshold probabilities. A smaller difference between the two curves indicates fewer false positives and higher screening efficiency.

As shown in Figure 3, a predictive nomogram was constructed based on the 11 variables screened by LASSO regression. As shown in Figure 4A, the AUC in the training set was 0.777 (95% CI: 0.727–0.826), and the lowest cutoff probability was 0.461, with a sensitivity of 0.819, a specificity of 0.582. The positive predictive value was 0.539; the negative predictive value was 0.846; the positive likelihood ratio was 1.959; the negative likelihood ratio was 0.311; and the F1 score was 0.651. The calibration curve indicated that the nomogram has a relatively desirable calibration (Figure 4B). The decision curve revealed a threshold probability interval for the net benefit (Figure 4C). At a threshold of 0.461, the model demonstrated a greater rate of net benefit when comparing the two extremes. In the clinical impact curve, the predicted risk was essentially the same as the actual risk when the cutoff probability was 0.461 (Figure 4D).

Assuming a male patient (0 points), occupation: retired (16.1 points), residence: urban (19.5 points), no osteoarthritis (0 points), no GA (0 points), myodynamia: 3 (18.2 points), HR: 85 (16.0 points), NEU: 64.3 (23.5 points), GLU: 9.41 (36.7 points), AST: 31 (69 points), and APTT 24.2 (70.7 points), the final total score was 269.7, corresponding to a threshold probability of 0.148. This probability was less than the threshold probability of 0.461. Hence, we believed that this patient would not develop SHS within the next 6 months.

### 3.4 Nomogram validation

**Figure 5:**
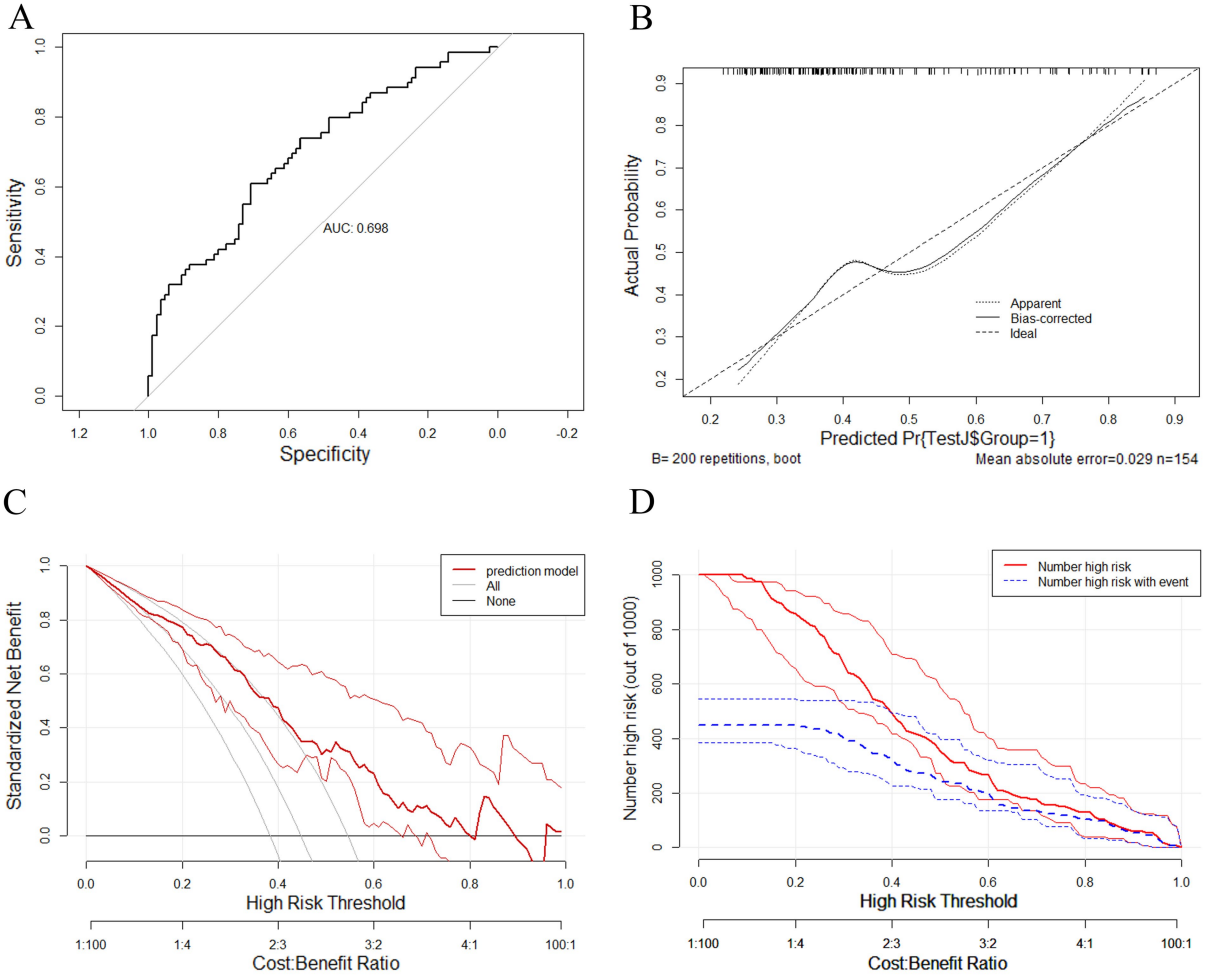
**(A)** Receiver operating characteristic curve of the nomogram for predicting post-stroke shoulder-hand syndrome in the validation set. **(B)** Calibration curve of the nomogram for predicting post-stroke shoulder-hand syndrome in the validation set. **(C)** Decision curve of the nomogram for predicting post-stroke shoulder-hand syndrome in the validation set. **(D)** Clinical impact curve of the nomogram for predicting post-stroke shoulder-hand syndrome in the validation set.

The nomogram was validated using data from the validation set. As shown in Figure 5A, the AUC in the validation set was 0.698 (95% CI: 0.615–0.781), with a sensitivity of 0.871, a specificity of 0.377. The positive predictive value was 0.531; the negative predictive value was 0.780; the positive likelihood ratio was 1.398; the negative likelihood ratio was 0.342; and the F1 score was 0.659 at a cutoff probability of 0.461. The calibration curves for the validation set similarly showed that the nomogram has a relatively desirable calibration (Figure 5B). The decision curve for the validation set showed that the model showed a greater net benefit rate when comparing the two extremes at a threshold of 0.461 (Figure 5C). In the clinical impact curve for the validation set, the predicted risk was generally consistent with the actual risk when the threshold was 0.461 (Figure 5D).

## 4 Discussion

In the present study, the incidence of SHS in patients with IS was 39.5%, which is consistent with previously reported incidence rates (Anwer and Alghadir, 2020). LASSO regression analysis revealed that 11 factors could serve as predictive factors for the occurrence of SHS, namely sex, occupation, residence, osteoarthritis, GA, myodynamia, HR, NEU, GLU, AST, and APTT. The nomogram constructed on the basis of these predictive factors had an AUC of 0.777 in the training set and 0.698 in the validation set. Based on the calibration curve, decision curve, and clinical impact curve, the model demonstrated relatively high accuracy. At the same time, the AUC of the validation set was lower than that of the training set, indicating that the model still had certain overfitting. Its performance still needs to be externally validated and improved.

Previous studies have primarily focused on the risk factors of SHS, while few studies construct predictive models for SHS. Regarding risk factors, Atas EU et al. have analyzed clinically relevant factors of CRPS (complex regional pain syndrome) in a retrospective cohort study. Their findings reveal that duration of hemiplegia, length of hospital stay, shoulder subluxation, soft tissue lesions, adhesion bursitis, spasticity, compression neuropathy, brachial plexus injury, protein-energy malnutrition, LRTI, urinary tract infection, depression, and coronary artery disease all significantly increase the incidence of CRPS (Altas et al., 2020). Regarding model prediction, in the study conducted by Yu et al. (2023), a total of 25 factors are included, and a random forest approach is employed to construct a predictive model for SHS. The constructed model has an AUC of 0.80, indicating that the model has good predictive ability. However, it is noted that this random forest study only collects data on 36 patients, which poses some challenges for the clinical application of the model. Furthermore, its sample size is small, which may affect the practical value of its predictive model. Another retrospective study on post-stroke hemiplegic shoulder pain (HSP) shows that shoulder subluxation, Brunnstrom stage (upper limb), hand edema, spasticity, and sensory disturbance are five independent predictors of HSP (Feng et al., 2022). Although it has developed a nomogram, the nomogram only predicts HSP. While HSP is a main symptom of stroke-related SHS, the two are not equivalent. Therefore, 514 patients were included in our study and a nomogram model was developed and validated. The purpose of this study was to help physicians identify potential patients with SHS and develop appropriate prevention strategies.

In our study, females were found to be a risk factor for developing SHS, which is consistent with previous findings (Sandroni et al., 2003). According to de Mos et al. (2008), the risk of CRPS is found to be significantly higher in women than in men, especially in women over 50 years of age. In our study, the mean age of the included patients was 66.85 ± 11.10 years old. Therefore, we believe there may be an association between women and an increased risk of SHS. This association may be explained by postmenopausal changes in sex hormone (such as estrogen) levels.

Our results indicate that place of residence and occupation are predictors of SHS. White-collar workers and civil servants among urban residents had a higher risk of developing SHS after stroke, possibly due to the lack of exercise. White-collar workers and civil servants generally sit at their desks for long hours and lack physical labor for a long time. Daviet et al. (2002) show that motor deficits are independent predictors of CRPS-I, which is also reflected in another predictive factor in our nomogram, namely, myodynamia. The model indicated that there was an increase in risk of SHS due to decreased myodynamia. Some studies have also shown that SHS is associated with impaired motor function after stroke. Gokkaya et al. (2006) report that severe dyskinesia and joint subluxation can increase the risk of CRPS-I. Braus et al. (1994) reveal that shoulder girdle muscle mild paralysis is a risk factor for SHS. Nadler et al. find a higher prevalence of shoulder pain in stroke patients with ≤ grade 2 shoulder abductor myodynamia (Nadler et al., 2020). These are consistent with our study. Furthermore, our analysis suggests that the high incidence of SHS may also be related to economic status and accessibility to healthcare. Rehabilitation resources are more concentrated in cities. Urban residents and civil servants/white-collar workers have easier access to rehabilitation medicine. They often have more opportunities for check-ups. At the same time, compared to rural residents and manual laborers, urban residents and civil servants/white-collar workers typically have stable jobs and social security. They are also more inclined to pursue higher-quality rehabilitation, actively seeking medical help rather than enduring the pain. These factors all contribute to the increased incidence.

Osteoarthritis and SA are also predictive factors for SHS. It is commonly believed that the development of SHS is associated with biomechanical changes in the shoulder on the hemiplegic side (Zyluk and Zyluk, 1999), including degeneration and wear of articular cartilage, fibrosis of the joint capsule and ligaments, muscle atrophy, and adhesions (Hawkes et al., 2012; Stanborough et al., 2022). These lesions can induce repeated micro traumas of the shoulder joint. These micro traumas can trigger abnormal regional sensory-sympathetic reflexes, leading to sensitization of the central nervous system, including the spinal cord and brainstem. As a result, pain perception and abnormal responses are further enhanced (Zyluk and Zyluk, 1999). An observational study by Li et al. (2022) shows that 69% of patients with SHS have combined supraspinatus tendinitis, and 66% have combined biceps longus tendinitis. They reveal that shoulder trauma may be one of the risk factors for SHS and recommend shoulder ultrasound for the prevention and diagnosis of SHS. In addition, osteoarthritis and GA can trigger local inflammatory reactions (Dalbeth et al., 2021; Sokolove and Lepus, 2013), resulting in an increase in inflammatory mediators (such as IL-1β, TNF-α, and IL-6) in joints and cartilage (Busso and So, 2010; Smith et al., 2004). Stroke itself can also cause acute inflammatory reactions, especially in IS, where damaged brain tissue releases large amounts of inflammatory mediators (Denes et al., 2010). These mediators (such as IL-6) enter the systemic circulation through the blood–brain barrier (Smith et al., 2004) and work together with local inflammatory mediators, increasing inflammation levels and the risk of SHS. Oral anti-inflammatory corticosteroids are also currently the only Class I recommended drug therapy for treating SHS (Harden et al., 2007). Both our results and previous studies suggest that osteoarthritis and gouty arthritis are associated with the occurrence of SHS and may be one of the predictors of SHS.

It was also found that a decrease in APTT levels can lead to a higher incidence of SHS in IS patients. APTT is a laboratory indicator for measuring blood coagulation ability. A low APTT indicates a shorter time for blood coagulation, indicating elevated coagulation function in the patient. The mechanism of SHS is related to local microcirculatory disorders. Lee et al. (2021) suggest that incorrect movement patterns in the early stages of stroke patients can induce shoulder injuries, resulting in impaired fluid circulation in the upper extremities. Zorowitz et al. (1996) suggest that central nervous system damage after stroke causes the loss of the muscle pump in the affected limb, leading to obstruction of venous return. A prolonged state of hypercoagulability induces damage to the vessel wall, affecting the elasticity and permeability of the vessel, which in turn influences the exchange of substances in and out of the vessel (Anderson and Weitz, 2011). Hypercoagulation also reduces blood flow velocity, impacting blood delivery and nutrient supply in microcirculation (Chiu and Chien, 2011). In addition, decreased blood flow may also cause the formation of blood clots (Gross and Aird, 2000), which may result in microcirculation obstruction and inflammatory reactions. Yu et al. (2023) reveal that the SHS group has higher levels of D-dimer based on the model, and suggest that anticoagulant therapy may be a method for preventing SHS. D-dimer is a product of fibrin degradation. An increase in its level usually indicates that the body is in a hypercoagulable state. This is consistent with our findings.

Given the favorable performance, the model is recommended in clinical settings. Operationally, if the nomogram shows that the risk of developing SHS in a patient is higher than the cutoff probability (0.461), it indicates a high risk of SHS. Clinicians need to provide early warning for such patients and implement timely preventative interventions. A randomized clinical trial by Hartwig et al. has demonstrated that wearing a shoulder subluxation orthosis significantly reduces SHS scores and prevents post-stroke SHS (Hartwig et al., 2012). A study by Kondo et al. has indicated that controlled passive activities and program management implemented by therapists, rather than arbitrary passive traction by patients or family members, can effectively reduce the incidence of SHS (Kondo et al., 2001). This finding suggests that high-risk individuals should be strictly prevented from performing inappropriate, excessive, or rough passive traction on their own. Matayoshi et al. administer weekly intramuscular injections of calcitonin to hospitalized patients with severe hemiplegia. Their results show a significant reduction in the incidence of SHS, and earlier injection is associated with the more pronounced preventative effects (Matayoshi et al., 2009). This result suggests that intramuscular calcitonin appears to inhibit the occurrence of SHS after stroke, especially in the early stages of stroke.

There are several limitations to this study. (i) This was a single-center retrospective cohort study. Due to limitations in previous medical records, there may be some variable selection bias and potential confounding factors in the data collection process. (ii) Although we included more medical records than previous studies, it is still limited compared to multicenter studies. A wider sample size for external testing is still needed to validate the reliability of the model in the future. (iii) We included 49 variables, which were richer than previous studies. Nonetheless, some imaging variables may still not be included, because many imaging examinations are expensive, and some patients have not undergone the examination. Moreover, there were inconsistencies in image parameters among some imaging reports. Therefore, we did not include some imaging factors that we considered valuable, such as shoulder MRI and musculoskeletal ultrasound. It is expected that future studies can make up for this deficiency. (iv) The model in this study is based on retrospective data from a rehabilitation center in China. There may be differences in case mix, stroke type, and other domains compared with other regions. Meanwhile, different regions and health systems have different allocations of rehabilitation resources and intervention programs. All of the above differences may affect the predictive performance and generalizability of the model. Therefore, before using this model in routine clinical practice, it is necessary to conduct external validation and model calibration in different regions, different medical levels, and multi-center populations to confirm its robustness and generalizability.

Despite its limitations, our study still holds significant clinical significance. This study is the first to use a nomogram to construct a post-IS SHS model. The nomogram was also observed to reflect reality well by various tests in the training and validation sets. The ROC analysis results indicated that the nomogram has favorable predictive performance. This model provided a tool for clinically predicting the risk of SHS in patients with IS and developing preventive programs for SHS. Therefore, our study is meaningful.

## 5 Conclusion

The nomogram constructed on the basis of common interpretable clinical features is a simple and reliable model for predicting the risk of SHS in patients with IS. The risk of SHS within 6 months after stroke is relatively well predicted. The nomogram may be used to early predict the risk of SHS in patients with IS. Accordingly, effective preventive protocols can be developed in a timely manner, thus improving the prognosis of patients. Given some limitations of our study, the accuracy of this prediction model remains to be validated and improved with a wider sample size in the future.

## Data Availability

The original contributions presented in the study are included in the article/supplementary material, further inquiries can be directed to the corresponding authors.
